# Burnout and Doctors: Prevalence, Prevention and Intervention

**DOI:** 10.3390/healthcare4030037

**Published:** 2016-06-30

**Authors:** Shailesh Kumar

**Affiliations:** Health Waikato, Waikato Clinical Campus, University of Auckland, Private bag 3200, Hamilton 3200, New Zealand; Shailesh.kumar@waikatodhb.health.nz; Tel.: +64-7839-8899; Fax: +64-7839-8972

**Keywords:** burnout, workplace stress, compassion fatigue, work engagement, resilience

## Abstract

Doctors are exposed to high levels of stress in the course of their profession and are particularly susceptible to experiencing burnout. Burnout has far-reaching implications on doctors; patients and the healthcare system. Doctors experiencing burnout are reported to be at a higher risk of making poor decisions; display hostile attitude toward patients; make more medical errors; and have difficult relationships with co-workers. Burnout among doctors also increases risk of depression; anxiety; sleep disturbances; fatigue; alcohol and drug misuse; marital dysfunction; premature retirement and perhaps most seriously suicide. Sources of stress in medical practice may range from the emotions arising in the context of patient care to the environment in which doctors practice. The extent of burnout may vary depending on the practice setting; speciality and changing work environment. Understanding dynamic risk factors associated with burnout may help us develop strategies for preventing and treating burnout. Some of these strategies will be reviewed in this paper.

## 1. Introduction

Doctors are often exposed to high levels of stress in their day-to-day work and are at greater risk of experiencing mental disorders, substance abuse, suicide, and impairment in functioning. By virtue of their work they are exposed to a plethora of emotions, including a need to rescue the patient, a sense of failure and frustration when the patient's illness progresses, feelings of powerlessness against illness and its associated losses, grief, fear of becoming ill oneself or dying, facing uncertainty in clinical practice, dealing with sexuality, or a desire to separate from and avoid patients to escape these feelings [1,2]. These emotions, powerful in nature and capable of causing distress, arise from the doctor−patient relationship. Repeated exposure to these emotions and experience of distress do contribute to the high levels of stress doctors experience in their profession.

Doctors are exposed to stressors from emotions and situations that arise outside the doctor–patient relationship too. They have to work in an increasingly litigious and unforgiving environment [3]. Bureaucratic requirements imposed upon them are increasing and keep changing [4]. Medical knowledge is advancing rapidly and doctors have to constantly keep in touch with it [4]. These changes are often so rapid that by the time doctors have acclimatised with one change something else may come up or evolve. Healthcare resources are limited in most countries and the environment is unforgiving of mistakes. A recent publication by the World Medical Association observed [5]:

“Physicians in many countries are experiencing great frustration in practising their profession, whether because of limited resources, government and/or corporate micro-management of health care delivery, sensationalist media reports of medical errors and unethical physician conduct, or challenges to their authority and skills by patients and other health care providers”

(5, p. 114). [5]

Furthermore, doctors are finding themselves working in an environment or in roles for which they were not trained. Service delivery is changing from an office-based model to a population-based health model. Doctors have to fulfil administrative duties, such as dealing effectively with workforce issues, often in addition to their significant clinical commitments [4]. Fulfilling unaccustomed tasks creates stressors, and constantly changing work environments predispose doctors to high levels of stress. Not uncommonly, doctors deal with these routine stressors by engaging in emotional withdrawal, social isolation and by denying the existence of problems [2]. Some of these strategies may be adaptive but pathological responses to chronic exposure to stress do occur which are explored further in this paper.

## 2. Impact of Chronic Exposure to Stress on Doctors

Experience of high levels of stress for protracted periods may have wide-ranging effects on doctors. Those who work with traumatised patients may experience compassion fatigue. Doctors experiencing compassion fatigue may experience intrusion, avoidance, and arousal that may occur even after exposure to one incident [6]. Two coping skills—sense of achievement and emotional disengagement, are said to protect against compassion fatigue, while prolonged exposure to traumatic materials, traumatic recollections, and life disturbances lead to the development of compassion fatigue. Most doctors aspire to demonstrate compassion for their patients and their work. They are likely to feel distressed if they have to practice in a compassion-depleted state.

Another consequence of chronic exposure to stressors is burnout. Burnout, a term in common usage, was first coined by Freudenberger [7] in 1974 to describe the emotional exhaustion experienced by workers in the public services. Most of the publications on burnout since its inception have targeted human service workers, acknowledging the unique pressures of utilising one’s self as the “tool” in face-to-face work, with needy, demanding, and often troubled clients [8]. Doctors fall in this category of professionals at high risk of experiencing burnout. Maslach and Jackson’s [9] conceptualised burnout as a three-dimensional construct consisting of Emotional Exhaustion, Depersonalisation and reduced Personal Accomplishment. Emotional Exhaustion (tiredness, somatic symptoms, decreased emotional resources and a feeling that one has nothing left to give to others) is used to describe a state of feeling emotionally overextended and exhausted by their work. Depersonalisation describes negative, cynical attitudes, and impersonal feelings towards clients which results in treating them as objects. Reduced Personal Accomplishments denote feelings of incompetence, inefficiency, and inadequacy. The higher the Emotional Exhaustion and Depersonalisation scores, and the lower the Personal Accomplishment score, the more the doctor could be suffering from burnout.

Exposure to severe and chronic stressors may also predispose doctors to a variety of mental morbidities and dysfunction including depression, anxiety, sleep disturbances and fatigue, broken relationships, alcohol and drug addictions, marital dysfunction, premature retirement and perhaps most seriously suicide [10,11]. As a group they have higher prevalence of depression and burnout than the general population and many other professional groups [3,12]. One interesting finding about depression in doctors is that many of the risk factors associated with depression in the general population are not applicable to them. Such risk factors include low socio-economic status, low education, unemployment, and being female [1]. Doctors experiencing burnout are also reported to be at greater risk of making poor judgment or errors in patient care, disengaging from work, demonstrate hostility toward patients, have diminished commitment and dedication to productive, safe, and optimal patient care as well as have difficult relationships with co-workers [10].

It is also possible that compassion fatigue, burnout and various other forms of mental morbidities in the face of chronic exposure to stress may be part of a spectrum and the manifestations may be related to each other. A recent study did find strong correlation between compassion fatigue and burnout (r = 0.769, *p* < 0.001) among family physicians [13]. This paper will focus on burnout among doctors and will not address the issue of burnout among medical students or junior doctors who may be exposed to different types of work environment-related stressors than their senior counterparts. This paper will also focus on strategies for preventing or treating burnout among doctors as opposed to reducing or managing stress among the medical profession notwithstanding the fact that chronic exposure to stress may lead to burnout among some doctors. It is recognised not every doctor exposed to chronic stress experiences burnout.

## 3. How Big is the Problem?

Estimate of burnout in doctors often yields high figures and varies between countries, across time, specialities or sector of work, i.e. public/private or rural/urban. This variation is understandable and expected because burnout is related to stressors arising from work environment and work environment is influenced by these variables. A study from rural British Columbia reported that 80%of physicians suffered from moderate to severe Emotional Exhaustion, 61% suffered from moderate to severe Depersonalisation, and 44% had moderate to low feelings of Personal Accomplishment [14]. A more recent study of US physicians [15] found 46% of the respondents had at least one symptom of burnout. European General Practice Research Network Burnout Study Group, on the other hand, found that, while 12% of participants suffered from burnout in all three dimensions, 43% scored high for Emotional Exhaustion, 35% for Depersonalisation, and 32% for low Personal Accomplishment [16]. In the United Kingdom, approximately one-third of the physicians had features of burnout [17], which are comparable to studies from Arab countries like Yemen, Qatar, and Saudi Arabia [18,19,20].

Not only may the overall prevalence of burnout among doctors vary between countries, but the three dimensions of burnout may also vary. A recent meta-analysis [21] found doctors in the USA experienced lower levels of Emotional Exhaustion (EE) than their counterparts in Europe where quality, safety culture and career development opportunities were strong. American doctors experienced higher levels of EE when faced with work life conflict and if they used ineffective coping. European doctors with positive work attitudes, on the other hand, experienced lower EE levels and possibly depersonalisation (DP) than their American counterparts [21]. This systematic review by examining the impact of various work environment-related stressors (such as quality, safety culture) or personal attributes (such as work−life conflict or positive work attributes) on the three dimensions of burnout highlighted the need to study burnout in the context of these stressors. This is an important issue as neither burnout as a phenomenon nor the preventive or intervention strategies are worth investigating in isolation.

There is also some evidence to suggest prevalence of burnout may be increasing in certain countries. An American study observed “*Burnout and satisfaction with work-life balance in US physicians worsened from 2011 to 2014. More than half of US physicians are now experiencing professional burnout*” [15]. This study identified the following specialities with the highest prevalence of burnout in 2014: urology (63.6%); physical medicine and rehabilitation (63.3%); family medicine (63.0%); radiology (61.4%); orthopaedic surgery (59.6%); dermatology (56.5%); general surgery subspecialties (52.7%); pathology (52.5%); and general paediatrics (46.3%). Different disciplines and specialities may respond differently to environmental stressors. A study [21] found outpatient specialities experienced higher levels of EE than inpatient specialities when organisation structures were constraining, whereas EE levels decreased in the former specialities when autonomy was present. Intervention strategies for burnout may therefore need to consider this trend as one speciality may respond to one set of factors such as autonomy whereas others may respond to loosening of organisational constraints. Caution does need to be exercised in drawing far-reaching conclusions from these studies which have reported varying prevalence across countries, specialities and settings because they have largely reported associations and not causation. Understanding risk factors that are associated with burnout may help develop preventative and therapeutic strategies against burnout among doctors.

## 4. Risk Factors for Burnout among Doctors

It is important to recognise that burnout manifests in the context of work stress. Several positive aspects of the work environment are thought to lower levels of stress for staff; however, the reverse corollary may also be true, i.e. when these positive attributes are absent, stress levels and therefore risk for burnout may increase for doctors working in poorly functioning organisations. Attributes of a positive work environment include organisational functionality (e.g., internal communication systems able to give the right information to the right people at the right time); individual satisfaction, (e.g., whether individual professionals feel supported by the management, and feel appreciated from patients); family-work balance, (e.g., provision of kindergarten services and reduction of work-related calls); opportunities for professional development for staff; and competent leadership [22]. Doctors working in environments where these positive attributes are absent or less prevalent may experience higher levels of stress and be at risk of experiencing burnout. Work environments where attention to excessive workload, long work hours, fatigue, emotional interactions, cognitive demands from the nature of medical practice, restricted autonomy and impact of structural and organisational changes on clinical practice is not paid fosters burnout [23]. Significance of work environment was further highlighted by another study that found organisational factors, as opposed to illness severity of patients in an emergency department, were strongly associated with a higher level of burnout. Factors such as impaired work relationships with colleagues were found to be independently associated with higher burnout scores, whereas improved relationships with chief nurses and nurses were associated with a lower burnout score [24].

While most people working in poorly functioning organisations may be exposed to high levels of stress putting them at greater risk of experiencing burnout, certain demographic factors may accentuate the risk for burnout. These include young age, female gender, negative marital status, long working hours and low levels of job satisfaction [25]. Being mindful of these risk factors may enable service providers and leaders to provide more support for doctors at greater risk and assist in screening “at risk” population. The scope of manipulating these risk factors for modifying the onset of burnout is limited and certainly not always easy. For instance, if young age is an identified risk factor for burnout, waiting to become older may not be an appropriate response. Reports of association between burnout, gender and negative marital status on the other hand have the potential to encourage discriminatory employment practices. There is also some evidence to suggest the association between gender and burnout may vary among countries. Publications from the USA have reported higher prevalence of burnout among female doctors [26], whereas European studies have reported the opposite trend, i.e. higher prevalence among men [16]. Such variation in risk factors that are derived from studies of correlation is not uncommon.

Several dynamic or modifiable contributors to doctor burnout have been identified. Interference with family life is often cited as a significant contributor toward burnout among doctors. A study of medical specialists reported that interference of work on home life (odds ratio 1.54, 95% CI 1.35–1.76) and not being able to live up to one’s professional standards (odds ratio 1.57, 95% CI 1.37–1.80) were most related to stress. The study also found that feeling poorly managed and resourced (odds ratio 2.07, 95% CI 1.76–2.43) diminished job satisfaction [26]. Job satisfaction is an interesting variable that can affect burnout among doctors, though the relationship between the two appears controversial. Some studies have reported a positive correlation between high levels of burnout and high levels of job satisfaction [27,28,29,30]. Others have found low levels of job satisfaction may be associated with high levels of burnout [31], and yet there is some evidence to suggest the relationship between specific dimensions of burnout and job satisfaction may change over time [32]. An American study of physicians from all speciality disciplines (n = 7288) compared burnout and job satisfaction with a probability-based sample of the general US population and found satisfaction with work−life balance varied with speciality. Highest rate of satisfaction was reported by physicians practising dermatology, general paediatrics, and preventive medicine, whereas physicians practising general surgery, and obstetrics/gynaecology had the lowest rates. Interestingly, the study also found general surgery and internal medicine—despite having the lowest rates of satisfaction with work−life balance—had below-average burnout rates, whereas specialities with high burnout rates (like neurology) were not necessarily those least satisfied with work−life balance [33]. Such factors as achieving work−life balance or enhancing job satisfaction on the other hand may be achievable and could counter burnout. Understanding such dynamic risk factors therefore may help us develop more robust intervention strategies against burnout and clearly are worthy of further research. Different levels of stress and sources of stressors are reported among doctors working in rural and urban settings. A study of 1400 general practitioners working in rural Australia reported manageable on-call arrangements, followed by professional support, and variety in clinical work were the top factors determining retention in rural and remote Australia, while proximity to a city or large regional centre was the least important factor [34]. The investigators did not specify how their sample was selected, stratified, nor how the 50% response rate was obtained. Their subjects were asked to identify six factors that they found stressful, but the authors did not describe how or why those particular factors were chosen. The study did include social and professional factors, but inexplicably ignored economic factors. Similarly, Hayes and colleagues [35] reported perceived problems with secondary education for children, poor housing, personality clashes with colleagues, constancy of after-hours work, difficulty in obtaining locum relief, limited access to continuing medical education, bureaucratic requirements, and family pressures as significant reasons for leaving rural areas. Both studies had identified stressors that were significant enough to prompt doctors to leave rural areas. Efforts to retain doctors in rural areas will need to consider these stressors.

## 5. Prevention Strategies against Burnout among Doctors

Many of the risk factors for burnout among doctors are static in nature or are difficult to address through intervention strategies. In general, three levels of change are recommended in order to reduce the risk of burnout: (1) modifying the organisational structure and work processes; (2) improving the fit between the organisation and the individual doctor through professional development programmes so that better adaption to the work environment occurs; and (3) individual-level actions to reduce stress and poor health symptoms through effective coping and promoting healthy behaviours [21]. Some of these strategies considered effective for preventing burnout among doctors will be discussed here.

Work engagement is being increasingly identified in occupational health psychology literature as preventative against burnout [36] as it has the potential to improve the fit between the organisation and the employee. It is a positive attribute, and in many ways just the opposite of burnout, characterised by vigour, dedication, and absorption, each of which has been operationally defined [37]. High levels of mental energy, persistence, and resilience are considered to characterise vigour, whereas a sense of significance, enthusiasm, inspiration, pride, and challenge characterise dedication. Absorption is characterised by being engrossed in work, which gives rise to the feeling that time at work passes quickly. Learning about factors which enhance vigour, dedication, and absorption among doctors will help us learn about work engagement in this group. It has generally been recognised that engaged workers have high levels of energy and identify strongly with their work [36]. Whether doctors who are better engaged with their work would be protected against burnout will need to be seen through future research.

Building resilience is often suggested as a preventative strategy against burnout among doctors [38,39]. At an individual level it can be seen as an attribute that may be effective in reducing effects of stress through effective coping and promoting healthy behaviours. Resilience is defined as “ability to bounce back or recover from stress” [37] or “the adoption of positive coping strategies, in times of change or adversity, to enable people to carry on in their jobs and lives” [38]. A Canadian study of physicians [40] identified resilience as a dynamic, evolving process of positive attitudes and effective strategies. The study found four main aspects of physician resilience: (1) attitudes and perspectives, which include valuing the physician role, maintaining interest, developing self-awareness, and accepting personal limitations; (2) balance and prioritization, which include setting limits, taking effective approaches to continuing professional development, and honouring the self; (3) practice management style, which includes sound business management, having good staff, and using effective practice arrangements; and (4) supportive relations, which include positive personal relationships, effective professional relationships, and good communication.

Deploying resilience-building strategies at an individual level in the effort to prevent burnout among doctors may be akin to building “herd immunity” through individual vaccination and yield greater reductions in stress levels among doctors. It is important to recognise focusing on resilience training of the individual doctor may distract attention from the work environment and organisational culture that causes burnout itself. No measures to prevent burnout will be effective unless attention is paid to enhancing a positive work environment. Isolated strategies directed at individual doctors may prove of limited benefit. A recent systematic review that examined correlates of physician burnout across regions and specialities identified that while it was important to reduce stress and poor health symptoms at the individual level through effective coping and promoting healthy behaviours that ultimately modify the organisational structure, work processes and improving the fit between the organisation and the individual physician were also crucial for preventing burnout [21]. A positive work environment is defined as one “*that attracts individuals into the health profession, encourages them to remain in the health workforce and enables them to perform effectively including professional development programmes to facilitate better adaptation to the work environment*” [22]. Key features of a positive work environment include where work−life balance is achieved by providing a family-friendly work environment and flexible working hours. Protection from exposure to occupational risks, enhancing job security, provision of childcare opportunities, compensation for reduced employment and maternity/parental leave were identified as attributes of work environment that prevent burnout.

## 6. Interventions for Burnout

Once burnout has set in, one has to look at therapeutic strategies. There is a shortage of well-designed studies on interventions for established burnout among doctors. There are anecdotal reports of a wide range of strategies being effective against burnout once it has set in. Such strategies include participation in panel and group discussions, conferences, and retreats without having to take time off; providing a list of resources to doctors including books, websites, and contact information for experts and workshop leaders who are trained in combating burnout; having professional body policy acknowledging the specific occupational stressors faced by physicians and encouraging physician self-care through proper rest and exercise, spending time with family and having a personal physician to assess well-being objectively; setting limits on hours and choosing a certain type of medical practice, being positive and maintaining a balance in life [11].

Stress management programmes are often recommended for managing burnout. A systematic review [41] found no evidence of effectiveness of brief stress management training interventions in reducing job stress for health workers. While this systematic review did not look at specific interventions for doctors, it did find low-quality evidence to support the effectiveness of stress management training of moderate intensity (defined as more than six hours contact over one month) in short-term reduction of job stress levels, but the beneficial effects diminished without booster sessions. The review found strong levels of evidence to support the effectiveness of intensive, long-term stress management training programmes in reducing workplace stress and risk of burnout among a wide range of health workers. This systematic review specifically examined the effectiveness of different strategies on reducing workplace stress. It is worth keeping in mind that workplace stress does not necessarily equate to burnout. Chronic exposure to workplace stress leads to burnout in some individuals, but not everyone exposed to chronic stress develops burnout. Therefore, simply reducing stress levels may not necessarily lead to reducing the risk of burnout. Factors such as personality traits and personal circumstances may determine whom among those exposed to chronic stress may experience burnout. Strategies found effective in reducing work-place stress may not necessarily and automatically reduce burnout unless these personal factors are evaluated and intervention strategies individualised to the person experiencing burnout.

Findings from a systematic review [42] may shed some light on the effectiveness of individualised intervention strategies against burnout. This systematic review [42] grouped intervention strategies against burnout into Person-directed (cognitive behavioural therapy, relaxation, music making, massage, and multi-component programmes) and Work-directed (attitude change and communication, support from colleagues, participatory problem solving and decision making, and changes in work organisation). The authors found limited evidence to support the efficacy of either Person- or Work-directed intervention strategies in reducing burnout in healthcare workers, and highlighted the need for good-quality intervention studies for burnout. Participation in “wellness programmes” was suggested to be related to lower incidence of burnout among doctors [25] although another systematic review that cautioned no causal relationship between participation in wellness programmes with lower incidence of burnout in doctors could be established.

There is some evidence to suggest participation in a mindful communication programme may be associated with short-term and sustained improvements in burnout among doctors. An American study examining the effect of an intensive educational programme consisting of an eight-week intensive phase (2.5 h/week, 7 h retreat) followed by a ten-month maintenance phase (2.5 h per month) teaching in mindfulness, communication, and self-awareness found improvements in mindfulness to be associated with significant improvements in all three dimensions of burnout (emotional exhaustion, 26.8 to 20.0; = −6.8; 95% CI, −4.8 to −8.8; depersonalisation, 8.4 to 5.9; = −2.5; 95% CI, −1.4 to −3.6; and personal accomplishment, 40.2 to 42.6; = 2.4; 95% CI, 1.2 to 3.6) [43].

Based on the evidence reviewed, one could speculate an intense stress management programme with booster sessions delivered over a longer period may yield longer-lasting results among those experiencing burnout. Alternatively, mindfulness-based strategies may be promising. Intervention studies comparing the efficacy of long- and short-term programmes of varying intensities against burnout among doctors are needed.

## 7. Conclusions

Burnout among doctors is a global phenomenon. By virtue of their profession, doctors are a vulnerable group for experiencing burnout. Burnout among doctors can lead to poor quality of care delivered to patients, increased medical errors and poor retention, in addition to poorer health outcome. Enhancing resilience and work engagement at an individual doctor level while also creating a positive work environment that helps doctors achieve work−life balance and enjoy job security, and provides a family-friendly work environment may help prevent burnout among doctors. Once burnout has set in, there is limited evidence to support the usefulness of modalities such as cognitive behavioural therapy, relaxation, music, or creating a positive work environment. Prevention appears to be, once again, far more beneficial than treatment when it comes to burnout.
